# Study on the Intervention Effect of Qi Gong Wan Prescription on Patients with Phlegm-Dampness Syndrome of Polycystic Ovary Syndrome Based on Intestinal Flora

**DOI:** 10.1155/2020/6389034

**Published:** 2020-09-29

**Authors:** Ning Zhang, Chang Li, Ying Guo, Hai-cui Wu

**Affiliations:** ^1^Shandong University of Traditional Chinese Medicine, Jinan 250355, China; ^2^Affiliated Hospital of Shandong University of Chinese Medicine, Jinan 250011, China

## Abstract

**Objective:**

This was a population-based cohort study, to compare the type and structure of intestinal flora in patients with polycystic ovary syndrome (PCOS) with phlegm-dampness syndrome, nonphlegm-dampness syndrome, and normal population. Besides, changes in the intestinal flora and the clinical curative effects of Jiawei Qi Gong Wan on phlegm-dampness syndrome in patients with polycystic ovary syndrome (PCOS) were evaluated. *Patients and Methods*. A total of 22 patients with PCOS with phlegm-dampness syndrome, 21 PCOS patients with nonphlegm-dampness syndrome, and 20 control volunteers were selected for this study. The general index, sex hormone index, fasting blood glucose (FPG), and serum fasting insulin (FINS) were determined in each of the groups. The intestinal flora of each group was determined by the 16s rDNA high-throughput sequencing technique. Besides, the PCOS with phlegm-dampness syndrome group was treated with Jiawei Qi Gong Wan, and the changes in TCM syndrome, sex hormone index, BMI, WHR, FPG, FINS, HOMA-IR, and intestinal flora were determined before and after treatment.

**Results:**

PCOS patients with phlegm-dampness syndrome showed characteristics of obesity and insulin resistance. There were several differences in both structure and function of the intestinal flora between PCOS patients with phlegm-dampness syndrome, PCOS patients with nonphlegm-dampness syndrome, and the control group. An imbalance in the intestinal flora may be a key factor in the pathogenesis of PCOS with phlegm-dampness syndrome and also has a specific influence on glucose and lipid metabolism, obesity, and the menstrual cycle in PCOS patients with phlegm-dampness syndrome. Besides, the imbalance is associated with a decrease in the number of butyrate-producing bacteria, an increase in the number of lipopolysaccharide-producing bacteria, and an increase in proinflammatory bacteria. The intestinal flora in PCOS patients with phlegm-dampness syndrome was found to be linked to obesity, impaired glucose tolerance, and hyperandrogenemia. Treatment with Jiawei Qi Gong Wan was found to increase the diversity of intestinal flora, increase the number of intestinal probiotics, and improve the structure and functional genes of intestinal flora which improved the state of insulin resistance, regulated endocrine metabolism, and improved the overall symptoms.

**Conclusions:**

Intestinal flora imbalance is a key factor in the pathogenesis of PCOS with phlegm-dampness syndrome. Besides, Jiawei Qi Gong Wan improves insulin resistance which is linked to the status of the intestinal flora in PCOS patients with phlegm-dampness syndrome.

## 1. Introduction

Polycystic ovary syndrome (PCOS) is a common endocrine disorder among women aged between 18 and 44 years of age. Clinically, menstrual abnormalities, hyperandrogenism (HA), obesity, rare ovulation or anovulation, and insulin resistance (IR) are some of the main features of PCOS. Besides, PCOS are associated with a series of metabolic disorders including type 2 diabetes, dyslipidemia, concentric obesity, and cardiovascular disease, among others. The prevalence of PCOS among women of childbearing age is about 5% to 10% [1]. Recent societal and economic developments have rapidly changed people's lifestyles, and together with environmental factors and other external factors, these have caused an increase in the incidence of polycystic ovary syndrome. In Chinese medicine, there is no special record of PCOS. However, due to diversity in clinical manifestations, TCM discussions are scattered concerning the symptoms of PCOS such as late menstruation, metrorrhagia, amenorrhea, dysentery, and infertility, and dialectical treatment is based on the symptoms. According to traditional Chinese medicine, the dysfunction of qi, blood, and body fluid leads to the endogenesis of phlegm and dampness, and the accumulation of phlegm aggravates the abnormal function of qi, blood, and body fluid. Ye Tianshi, an ancient Chinese physician, put forward the importance of physical factors in disease differentiation and syndrome differentiation. He further noted that dampness is a yin pathogenic factor; it tends to descend and attack the yin body sites; and it is turbid, sticky, and lingering and tends to affect first the lower part of the body, causing abdominal fullness. The phlegm-dampness constitution is one of the main pathogenesis common in obese people.

Clinical studies have shown that most of the common pathological features in obese people are also associated with phlegm-dampness constitution. As the “human second genome,” the intestinal flora genes have become a research hotspot in recent years. Numerous studies have shown that the intestinal flora is closely related to endocrine and metabolic diseases such as insulin resistance, obesity, and diabetes. Once the flora structure changes and the intestinal flora is imbalanced, it is likely to cause disorders associated with the body's metabolism; however, its specific mechanism of action is not completely clear.

The current study compared the intestinal flora spectrum, endocrine, and metabolic indicators of PCOS patients with phlegm-dampness syndrome and nonphlegm-dampness syndrome and a control group. The study also compared the differences in intestinal flora structure in each group based on the indicators of obesity and insulin resistance. To determine the predominant flora in PCOS patients with phlegm-dampness syndrome, this study established a diagnostic model based on the intestinal microbiota and used it to observe the changes in the intestinal flora and the clinical efficacy following treatment with Jiawei Qi Gong Wan. Besides, the study explored the mechanism of action and therapeutic target of Qigong Wan prescription.

## 2. Materials and Methods

### 2.1. Participants

This study was approved by the Ethics Committee of the Reproductive and Genetic Center of the Affiliated Hospital of Shandong University of Traditional Chinese Medicine. Before the commencement of the study, the research purpose, content, and research process was explained in detail to each participant, who was required to sign a written informed consent. The sample size of this study was calculated using the sample size estimation formula of the multisample average. It was calculated based on the proportion of the difference in the intestinal flora among the groups, and the power of this study was 0.8. The finding showed that each group needed 22 cases. The researchers' tried to collect a sample size close to the expected sample size. Due to a large number of testing items, relatively few complete testing cases were considered, but the data were considered valid and reliable. However, due to the small sample size in this study, type 1 error could not be excluded and might have occurred during statistical analysis.

A total of 63 patients with PCOS and normal endocrine function were selected from the Reproductive and Genetics Center of the Affiliated Hospital of Shandong University of Traditional Chinese Medicine between December 2018 and October 2019. The patients included 22 PCOS patients with phlegm-dampness syndrome, 21 PCOS patients with nonphlegm-dampness syndrome, and 20 patients in the control group. The diagnosis of PCOS was done according to the Rotterdam criteria of 2003. The diagnosis excluded the following: patients who had received hormonal drugs within 3 months before the study; patients with physiological menstruation and amenorrhea; patients with serious cardiovascular and cerebrovascular diseases, such as hypertension and coronary heart disease; patients with liver and kidney diseases; patients with mental disorders; patients with acute infection of genitourinary system; patients with sexually transmitted diseases; patients with infectious diseases and patients with hematological diseases; patients with abnormal endocrine functions such as thyroid, adrenal gland, and pituitary gland, including hyperthyroidism, adrenocortical hyperplasia, adrenocortical hypofunction, pituitary tumor, Sheehan's syndrome, and diabetes; patients with reproductive system tumors; patients with organic diseases of reproductive organs; patients with immunodeficiency or autoimmune diseases; patients with digestive tract diseases or previous digestive tract surgery; patients using acid-making agents, probiotics, corticosteroids, antibiotics, or antimicrobial agents that may affect the human intestinal flora within one month; and patients who are allergic to certain drugs.

The standard of syndrome differentiation of phlegm-dampness syndrome is based on the textbook “Gynecology of Traditional Chinese Medicine” edited by Luo Songping, “TCM syndrome differentiation and treatment system” edited by Fangnan [2], and “Classification and judgment of TCM constitution” published by Chinese Society of Traditional Chinese Medicine in 2009. The criteria for TCM syndrome differentiation of phlegm-dampness syndrome are as follows: main symptoms included delayed or gradual amenorrhea or long-term marriage infertility; concomitant symptoms included obesity or mental fatigue and drowsiness; the fullness of chest and abdomen; a small amount of menstrual period: light dark or light red menstrual period; sticky phlegm; easy nausea and vomiting; the quantity of sticky and greasy stool; thin or turbid stool; tongue and pulse: light, fat, and toothed tongue, greasy moss, and pulse sinking or slippery slow. The main certificate must have been available, and the patients were required to meet 3 of the remaining items, combined with the tongue, to make a diagnosis.

Among the 63 patients included in this study, 2 patients in the PCOS with phlegm-dampness group showed poor compliance and did not take drugs following the regulations, and 1 PCOS patient with nonphlegm-dampness group had missing examination results and withdrew from the study. Therefore, 20 patients in the PCOS with phlegm-dampness group, 20 patients in the PCOS with nonphlegm-dampness group, and 20 patients in the endocrine normal group were included in statistics. All the patients had no guidance on lifestyle adjustment.

### 2.2. Anthropometric and Metabolic Parameter Measurements

Detailed medical history and history for physical examination of the patients were collected at the initial diagnosis. Patients who met the inclusion criteria were given informed consent to sign. Basic information such as the age of PCOS patients and healthy control groups were collected, and the subjects' height (m), weight (kg), waist (cm), and hip circumference were measured by a fixed tester using the same measuring tool. The participants were required to empty their bladder and be on an empty stomach (cm), and their body mass index (BMI) (BMI = weight (kg)/height (m)^2^) and waist-to-hip ratio (WHR) (WHR = waist/hip) were calculated. All the subjects were required to stop taking any drugs that affected their reproductive endocrine and metabolism, three months before the study commenced. Fasting venous blood (2 ml) was drawn from the subjects, following 8–12 hours fast during the early follicular phase (days 2–4 of menstruation) or amenorrhea (no dominant follicles under B-mode ultrasound) and a 30 minutes rest. The blood samples were stored at room temperature and centrifuged at 4000 rpm for 6 minutes; the serum was transferred into a 1.5 mL EP tube and immediately stored in a −80°C refrigerator for future use. Detection of serum follicle-stimulating hormone (FSH), luteinizing hormone (LH), prolactin (PRL), estradiol (E2) and progesterone (P) total testosterone (T), fasting plasma glucose (FPG), and fasting insulin (FINS) was performed by chemiluminescence. The homeostasis model assessment (HOMA) was used to calculate the insulin resistance index (HOMA-IR) (HOMA-IR = fasting blood glucose (mmol/L) × fasting insulin (mU/L)/22.5) HOMA-IR > 2.77, which was used as the diagnostic criteria for PCOS with IR [3]. The larger the HOMA-IR value, the more severe the IR. Stool samples were collected using the following method: dry ice and foam boxes were prepared before sampling; fresh stool samples were collected from patients in the PCOS with phlegm-dampness group, PCOS with nonphlegm-dampness group, and the control group. The subjects were instructed to urinate before defecation to prevent urine from contaminating the stool samples as well as spread several layers of straw paper in the urinal to prevent water from contaminating the stool samples. A 5 ml disposable sterile fecal collector was used to collect 2-3 g of fresh stool sample and immediately packed it into a foam box containing dry ice and stored it in a −80°C refrigerator for 2 h. The patients in the PCOS with phlegm-dampness group were administered with the Jiawei Qi Gong Wan treatment for 2 months, after which samples were collected and frozen.

### 2.3. Treatment Method

The PCOS phlegm-dampness group was treated with Jiawei Qi Gong Wan, which was provided by the Affiliated Hospital of Shandong University of Traditional Chinese Medicine. The medicine composition is as follows: 12 g Zhebei, 12 g Xuanshen, 12 g Jineijin, 12 g Huanglian, 12 g Jiaoshanzha, 12 g Baizhu, 12 g Cangzhu, 12 g Xiangfu, 9 g Banxia, 12 g Chenpi, 12 g Shenqu, 6 g Gancao, 15 g Fuling, 12 g Chuanxiong, 15 g Tusizi, 12 g Duzhong, and 15 g Lianqiao. The treatment was administered based on the patient's clinical symptom. The treatment was taken from the fifth day of menstruation, decoction, 1 dose daily, warm twice daily, morning and evening, continuous for 2 months, with discontinuation of menstruation. If the menstrual period is 30 days, the urine will be tested for HCG. If it is negative, continue to take the medicine. If the menstruation continues, it will be rechecked every 5 days. If it is negative, continue to take the medicine. If it is positive, the experiment will be terminated. PCOS nonphlegm-dampness group: it is enough to adopt a treatment plan for the cause, and this study will not be followed up.

### 2.4. 16s rDNA Sequencing

Total genome DNA from the participants' samples was extracted using the CTAB method. DNA concentration and purity were monitored on 1% agarose gels. Based on the sample concentration, DNA was diluted to 1 ng/*μ*l using sterile water. The V3-V4 regions of the bacterial 16S rRNA gene were amplified using Universal primers 341F (CCTAYGGGRBGCASCAG) and 806R (GGACTACNNGGGTATCTAAT). PCR was performed in 30 *μ*L reactions using 15 *μ*L of Phusion® High-Fidelity PCR Master Mix (New England Biolabs), 0.2 *μ*M of forward and reverse primers, and about 10 ng of the DNA template. Thermal cycling conditions consisted of an initial denaturation at 98°C for 1 min, followed by 30 cycles of denaturation at 98°C for 10 s, annealing at 50°C for the 30 s, elongation at 72°C for 30 s, and final elongation at 72°C for 5 min. To detect the PCR products, a similar volume of 1 × loading buffer (contained SYB green) was mixed with the PCR products and electrophoresed on 2% agarose gel. The PCR products were mixed in equidensity ratios and purified using the GeneJETTM Gel Extraction Kit (Thermo Scientific). Sequencing libraries were generated using the Ion Plus Fragment Library Kit 48 rxns (Thermo Scientific), following the manufacturer's instructions. The library quality was assessed using a Qubit@ 2.0 Fluorometer (Thermo Scientific). Finally, the library was sequenced on an Ion S5 TM XL platform, and 400 bp/600 bp single-end reads were generated.

### 2.5. Statistical Analysis

#### 2.5.1. Clinical Data

SPSS statistical package 26.0 was used to perform all the statistical analyses for the clinical data, and data were expressed as means ± standard deviations. When comparing general data between multiple groups, those who meet the normal distribution use analysis of variance, pairwise comparison uses LSD test and nonnormal distribution, or uneven variance data use the nonparametric test (Kruskal–Wallis test). When comparing the general data before and after treatment, those who met the normal distribution and met the variance were tested by *t*, and those who did not meet the normal distribution were tested by nonparameters (Wilcoxon). Correlations between intestinal flora and clinical and biochemical variables were analyzed by Spearman correlation analysis. A *P* value < 0.05 was considered statistically significant.

#### 2.5.2. Bioinformatics Analysis

Single-end reads were assigned to each sample based on their unique barcode and truncated by cutting off the barcode and primer sequence. Quality filtering on the raw reads was performed under specific filtering conditions to obtain the high-quality clean reads according to the Cutadapt [4] quality-controlled process. The identified reads were compared with the reference database using the UCHIME algorithm [5] to detect and remove the chimera sequences [6]. Then, the Clean Reads were finally obtained. Sequences analysis was performed by Uparse software [7]. Sequences with ≥97% similarity were assigned to the same OTUs. The representative sequence for each OTU was screened for further annotation. To compute alpha diversity, we rarified the OTU table and calculated three metrics: Chao1 to estimate the species abundance, observed species to estimate the amount of unique OTUs found in each sample, and Shannon index. Rarefaction curves were generated based on these three metrics. QIIME calculated both weighted and unweighted UniFrac, which are phylogenetic measures of beta diversity. PCoA was used to obtain principal coordinates and visualize them from complex and multidimensional data. It takes a transformation from a distance matrix to a new set of orthogonal axes. The maximum variation factor was demonstrated by the first principal coordinate and the second maximum variation factor by the second principal coordinate and so on.

## 3. Results

### 3.1. Characteristics of the Study Subjects

The clinical and hormonal parameters are summarized in Table 1. The results showed that age, FSH, E2, P, and PRL did not differ between the groups. However, BMI, waist circumference, hip circumference, WHR, FPG, FINS, and HOMA-IR were higher in the PCOS with phlegm-dampness group than in the PCOS with nonphlegm-dampness group and the control group (*P* < 0.05). The LH, LH/FSH, and T were higher in both the PCOS with phlegm-dampness group and the PCOS with nonphlegm-dampness group than the control group, and the differences were statistically significant (*P* < 0.05). The LH in the PCOS with nonphlegm-dampness group was higher than that in the PCOS with phlegm-dampness group. However, there was no significant difference in LH, LH/FSH, and T levels between the two groups.

### 3.2. Diversity of Intestinal Microbial Communities

#### 3.2.1. Dilution Curve

**Figure 1:**
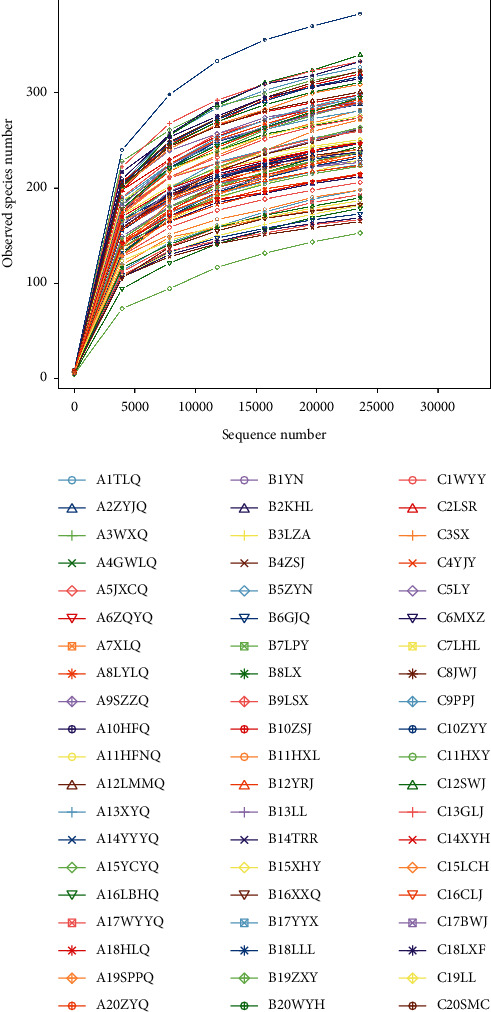
Dilution curve. Abscissa is the number of sequencing strips randomly selected from a sample, and the ordinate is the number of OTU that can be constructed based on the number of sequencing strips, and it is used to reflect the sequencing depth; different samples are represented by curves of different colors; with the expansion of sequencing quantity, the curve tends to flatten, indicating that the sequencing data are sufficient, and further sequencing does not increase species diversity.

The rarefaction curve is a common curve describing the diversity of samples within a group. When the curve is flat, this means that the amount of sequencing data is progressive and reasonable. More data only generate a small number of new species (OTUs). In this study, the analysis showed that the curve was gradually flat (Figure 1), indicating that the sample size met the sampling needs, the sequencing data volume was reasonable enough, and additional data volume would not result in more species differences.

#### 3.2.2. Alpha Diversity Analysis

**Figure 2:**
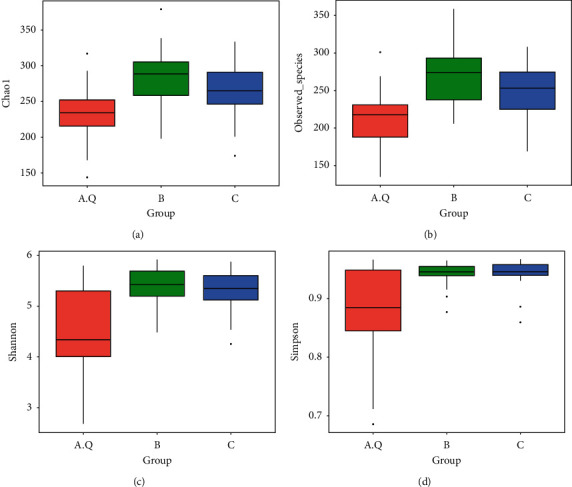
Box diagram showing the differences between groups of *α* diversity index. Horizontal axis: A.Q: PCOS with phlegm-dampness syndrome group intestinal flora; B: PCOS with nonphlegm-dampness syndrome group intestinal flora; C: control group intestinal flora. Vertical axis: *α* diversity index.

Alpha diversity is used to analyze the richness and diversity of microbial communities in a sample (within community) [8] and refers to the diversity within a specific area. *α* diversity index includes Shannon, Simpson, Chao1, ACE, goods_coverage, and PD_whole_tree. Chao1 is commonly used to calculate flora abundance. The larger the Chao1 index, the higher the species abundance, and the greater the total number of species. Shannon and Simpson calculate flora diversity. Chao1 index was B group > C group > A.Q group (Figure 2). The Chao1 index in the B and C groups was 0.1816, with no significant difference (*P* > 0.05). In the AQ-B and AQ-C groups, the *P* values were <0.0001 and 0.0081, and the difference was statistically significant (*P* < 0.01). Shannon index was in the B group > *C* group > AQ group. The *P* value of the Shannon index between BC groups was 0.6463, and the difference was not statistically significant (*P* > 0.05). The *P* values between AQ-B and AQ-C groups were <0.0001 and <0.0001, and the difference was statistically significant (*P* < 0.01). The *P* value of the Simpson index between the two groups of BC was 0.8337, with no significant difference (*P* > 0.05). The *P* values between the two groups of AQ-B and AQ-C were 0.0126 and 0.0071, respectively, and the difference was statistically significant (*P* < 0.05). The *P* value of observed OTUs between the two groups of B and C was 0.2157, and the difference was not statistically significant (*P* > 0.05). The *P* values between the two groups of AQ-B and AQ-C were <0.0001 and 0.0045, and the difference was statistically significant (*P* < 0.01). It can be seen that the species quantity (richness) of the AQ group community was less than that of the B and C groups (*P* < 0.01) and the B group > *C* group (*P* > 0.05). The diversity of the intestinal flora of the AQ group (quantity and Uniformity) was less than that of groups B and C (*P* < 0.05).

#### 3.2.3. *β*-Diversity Analysis

**Figure 3:**
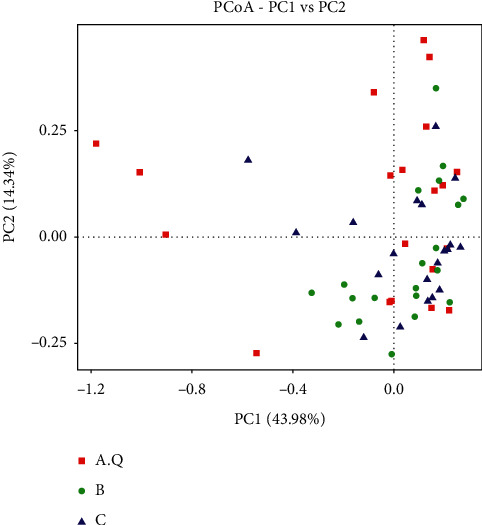
PCoA analysis of *β* diversity for weighted UniFrac indexes. The abscissa represents one principal component, the ordinate represents another principal component, and the percentage represents the contribution value of the principal component to the sample difference; each point in the figure represents a sample, and the samples of the same group are represented by the same color. The more similar in terms of colony composition, the closer the distance in the picture.

The function of PCoA analysis is to extract the most important elements and structures from multidimensional data through a series of eigenvalues and eigenvectors [9]. Therefore, the samples with high similarity in community structure are gathered together, and the samples with different community structures are far apart. The scattered points between the stool samples in the PCOS with phlegm-dampness syndrome group and the stool samples in the PCOS with nonphlegm-dampness syndrome group and the control group were relatively sufficient (Figure 3), and some could be distinguished. The scatter points between the stool samples of the PCOS with nonphlegm-dampness syndrome group and those of the control group were close and showed no significant difference in *β* diversity.

**Figure 4:**
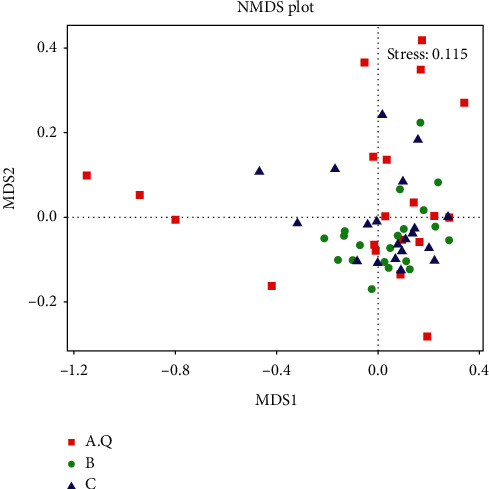
Each point in the graph represents a sample, the distance between the points indicates the degree of difference, and the samples of the same group are represented by the same color. When stress is less than 0.2, it shows that NMDS can accurately reflect the degree of difference between samples.

Nonmetric multidimensional calibration (NMDS) statistics are nonlinear models that can overcome the shortcomings of linear models (such as PCoA) and better reflect the nonlinear structure of ecological data [10]. The shorter the distance between the two points, the smaller the differences in community composition between the two. The results of NMDS analysis based on OTU levels are shown in Figure 4. The scatter separation between the stool samples in the PCOS with phlegm-dampness syndrome group and the stool samples in the PCOS with nonphlegm-dampness syndrome group and the control group stool samples was sufficient and distinguished. Partial scatter between PCOS with nonphlegm-dampness syndrome group and the control group stool samples was close, but no significant differences were found in aggregation and *β* diversity.

**Figure 5:**
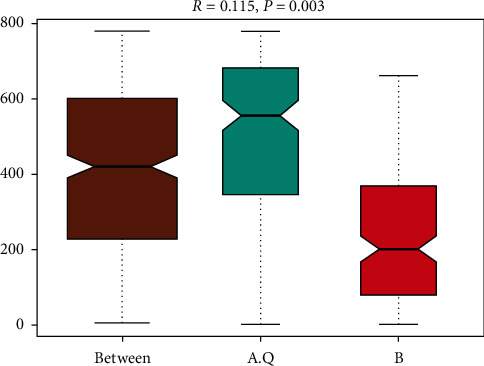
The ordinate is the rank of the distance between the samples, and the abscissa refers to the result between the two groups, and the other two are the results within the respective groups. The *R* value is between (−1, 1), and the *R* value is greater than 0, indicating that the differences between the groups are significant. The *R* value is less than 0, indicating that the difference within the group is greater than the difference between the groups. The credibility of the statistical analysis is expressed by *P* value, and *P* < 0.05 indicates significant differences.

Anosim analysis [11] is a nonparametric test used to determine if the differences between two or more groups are significant. Anosim analysis is based on the rank obtained by sorting the distance values between the two samples (between groups), and the comparison of any two groups can obtain three classified data and display the boxplot, as shown in Figure 5, *R* = 0.115, *P* = 0.003, *R* value greater than 0, and *P* < 0.05, indicating PCOS. There was a statistically significant difference between the PCOS with phlegm-dampness syndrome group and the PCOS with nonphlegm-dampness syndrome group.

### 3.3. Differences in Intestinal Microflora Composition

**Figure 6:**
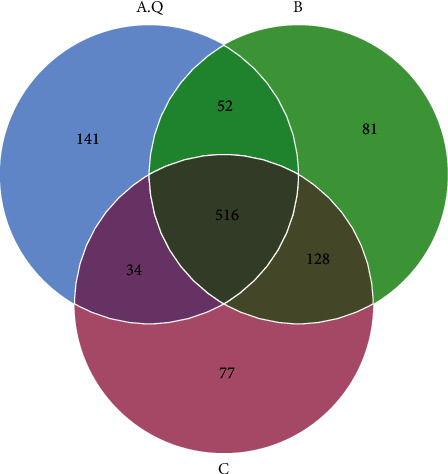
Venn plot diagram of the OTU distribution in the three groups. Each circle in the figure represents one (group) sample, and the numbers of the circle and the circle overlap represent the number of OTUs shared between the samples (group). The numbers without overlap represent the unique OTUs of the sample (group). A.Q: intestinal flora of PCOS with phlegm-dampness syndrome group; B: intestinal flora of PCOS with nonphlegm-dampness syndrome group; C: intestinal flora of the control group.

To reflect the similarity and overlap between the three groups of samples, the OUT Venn diagram is drawn in Figure 6. There were 516 OTUs distributed in the three groups of samples, and the number of overlapping OTUs between two groups was high, meaning that there were relatively high similarities. Besides, 141 OTUs were unique to the phlegm-dampness syndrome group, 81 OTUs were found to be distributed only in the nonphlegm-dampness syndrome group, and 77 OTUs were unique to the normal endocrine control group, suggesting that the three groups had differences between species distribution.

**Figure 7:**
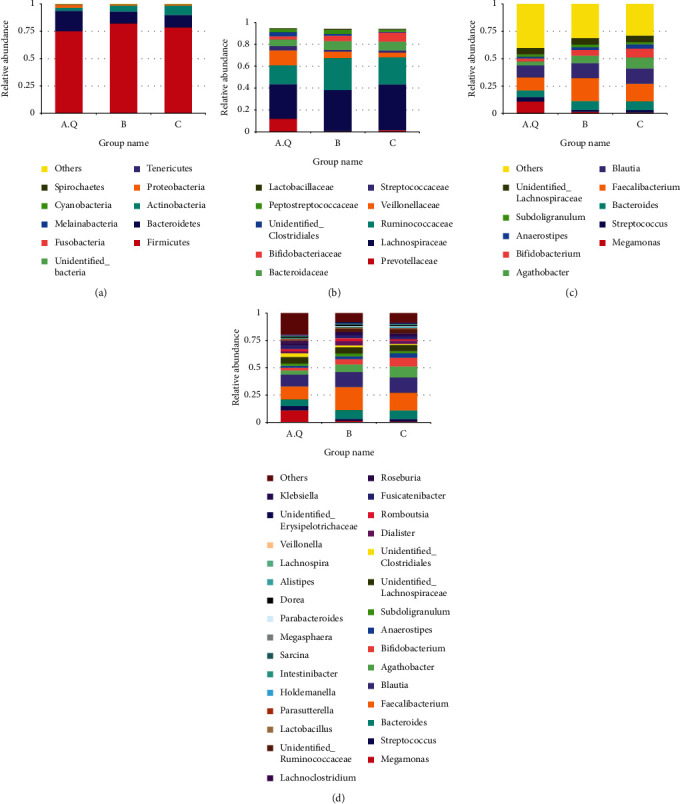
The abscissa is the group name; the ordinate represents the relative abundance. (a) Histograms of relative abundance of species at the phyla levels (top10) of the three groups; (b) histogram showing species relative abundance (top10) at the family level in the three groups; (c) histograms showing the relative abundance of species at the level of the genera (top 10) in the three groups; (d) histograms showing the relative abundance of species at the level of the genera (top30) in the three groups.

**Figure 8:**
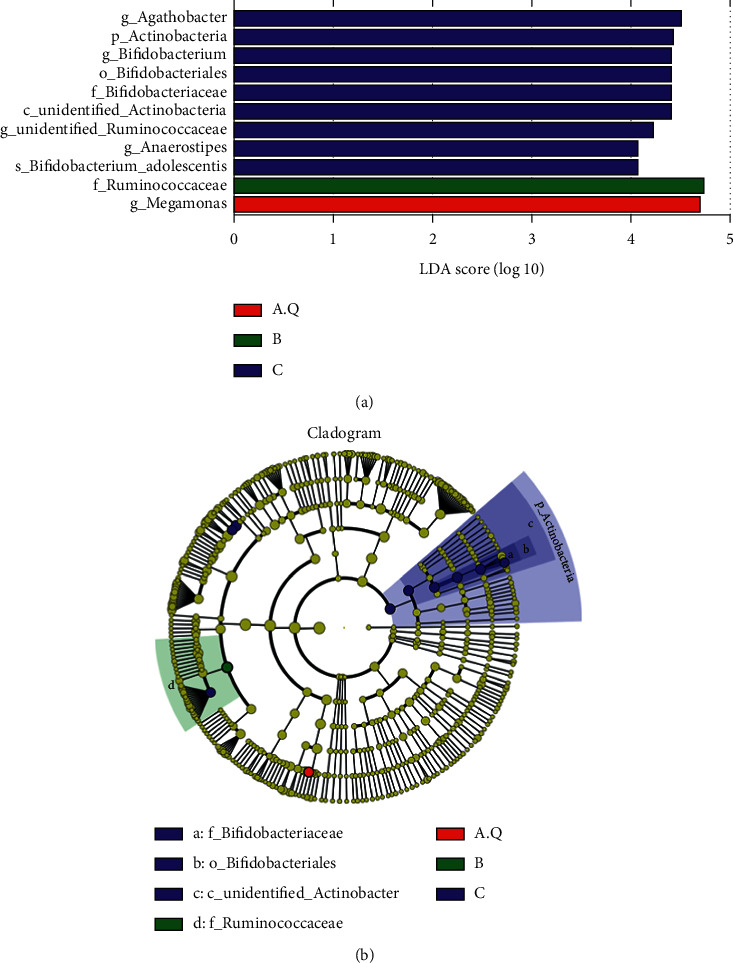
LDA value distribution histogram shows the species whose LDA score is greater than the set value (the default setting is 4), that is, the biomarker with statistical differences between groups. Species with significantly different abundances in different groups are shown, and the length of the histogram represents the magnitude of the impact of the different species (i.e., the LDA score). In the evolutionary branch diagram, the circle radiating from the inside to the outside represents the classification level from the door to the genera (or species). Each small circle at a different classification level represents a classification at that level, and the diameter of the small circle is proportional to the relative abundance. Coloring principle: the species with no significant difference is uniformly colored yellow, and the different species biomarkers follow the group for coloring. The red node indicates that the microbial group plays an important role in the red group, and the green node indicates that it plays an important role in the microbial groups. If a group is missing in the picture, it means that there are no significant differences in species in this group, and the group is missing. The names of the species are indicated by their English letters in the legend on the right.

As shown in Figure 7, at the phylum level, the histogram of the colony composition between the three groups showed that the three groups had similar colony composition, and the top 10 most abundant were as follows: Firmicutes, Bacteroidetes, Actinobacteria, Proteobacteria, Tenericutes, Fusobacteria, Melainabacteria, Cyanobacteria, and Spirochaetes. Among them, Firmicutes, Bacteroidetes, Actinobacteria, and Proteobacteria were the dominant phyla, accounting for more than 99% of the entire sequence. It can be seen that the proportion of Bacteroidetes, Bacteroides/Firmicutes, and Proteobacteria in PCOS with phlegm-dampness group was higher than that in PCOS nonphlegm-dampness group and control group. Besides, the proportion of Actinobacteria was smaller than that in PCOS with nonphlegm-dampness group and control group (Figure 8). Rank-sum test and LEfSe analysis showed that Actinobacteria were more enriched in the PCOS with phlegm-dampness syndrome group and PCOS with nonphlegm-dampness syndrome group than in the control group.

At the family level, the proportion of Prevotellaceae and Veillonellaceae was higher in the PCOS with phlegm-dampness group than in PCOS with nonphlegm-dampness group and control group. The proportion of Ruminococcaceae and Bifidobacteriaceae was smaller in the PCOS with phlegm-dampness group than in PCOS with nonphlegm-dampness group and control group in Figure 7. The rank-sum test and LEfSe analysis showed that, at the family level, Bifidobacteriaceae, Ruminococcaceae, and Bifidobacteriaceae were more enriched in the control group, and Ruminococcaceae was more enriched in the PCOS with nonphlegm-dampness syndrome group (Figure 8).

At the genus level, the proportion of Megamonas in the PCOS with phlegm-dampness group was larger than that in the PCOS with nonphlegm-dampness group and the control group. Besides, the proportion of Faecalibacterium and Bifidobacterium was smaller than that of the PCOS with nonphlegm-wet group and control group (Figure 7). The rank-sum check and LEfSe analysis showed that, at the genus level, there were significant differences among the three species: Agathobacter, Bifidobacterium, unidentified Ruminococcaceae, Anaerostipes, and Megamonas. Anaerostipes was more enriched in the control group than in the PCOS with phlegm-dampness syndrome group and PCOS with nonphlegm-dampness syndrome group. Megamonas was more enriched in the PCOS with phlegm-dampness syndrome group (Figure 8).

Bifidobacterium belongs to Actinobacteria; lactobacillus belongs to Firmicutes, Bacilli, and Lactobacillales. Figure 7 shows that, among the species with the highest abundance of 10 and 30 at the genus level, the content of Bifidobacteria in group A.Q was 2.8%, group B was 5.1%, and group C was 8.2%. As shown in Figure 7(d), at the genus level, the species with the highest abundance among the top 30 species was Lactobacillus and the content among the three groups was as follows: Lactobacillus: A.Q: 0.26%, group B: 0.68%, and group C: 0.37% (Figures 7(c) and 7(d)).

### 3.4. Correlation Analysis between Intestinal Flora and Clinical Indexes

**Figure 9:**
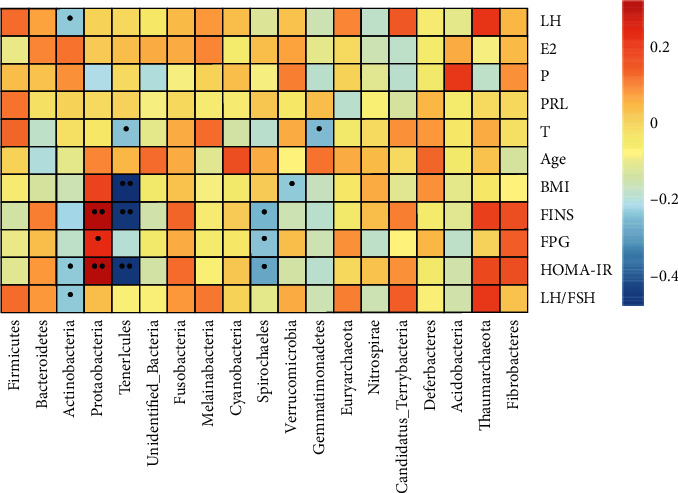
Spearman correlation analysis between environmental factors and intestinal flora: red represents positive correlation, and blue represents a negative correlation. Vertical is the environmental factor, horizontal is species information, and the corresponding value in the middle heat map is the spearman correlation coefficient *r* which is between −1 and 1; *r* < 0 is a negative correlation, *r* > 0 is a positive correlation, and ^*∗*^significance level at *P* value <0.05 and ^*∗∗*^*P* value <0.01.

Spearman rank correlation was used to determine the relationship between environmental factors and the richness of intestinal flora [12]. Actinobacteria was negatively correlated with LH, HOMA-IR, and LH/FSH. *P* value was <0.05. Proteobacteria was positively correlated with FINS, FPG, and HOMA-IR (*P* < 0.05). Tenericutes was negatively correlated with BMI, FINS, HOMA-IR, and T (*P* value <0.05). Spirochaetes was positively correlated with FINS, HOMA-IR, and FPG (*P* value <0.05) (Figure 9).

**Figure 10:**
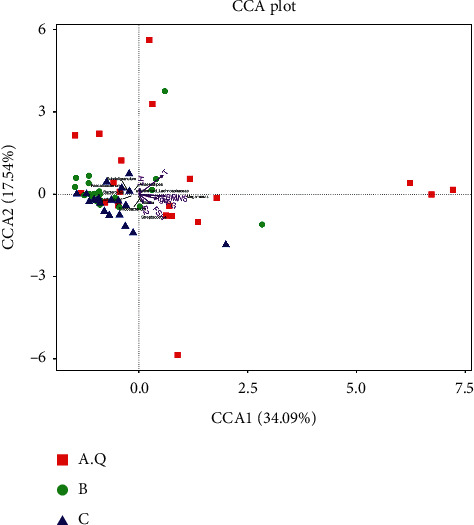
In the CCA ordination chart, environmental factors are generally expressed by arrows, and the length of the arrow line represents the degree of correlation between an environmental factor and community distribution and species distribution. The longer the arrowhead is, the greater the correlation, and vice versa. The angle between the arrow line and the sorting axis represents the correlation between an environmental factor and the sorting axis, the smaller the angle, the higher the correlation, and vice versa. When the angle between environmental factors is acute, it means that there is a positive correlation between the two environmental factors, and a negative correlation when the angle is obtuse.

All the samples were treated as a group, and canonical correlation analysis (CCA) between the clinical indicators and the relative abundance of the obtained genus-level flora was performed. CCA analysis can detect environmental factors, samples, and flora. The relationship between them or the relationship between two pairs [13] can obtain important environmental factors that affect the distribution of samples, as shown in Figure 10. The envfit function was used to test the significance of each environmental factor. The CCA envfit table shows the results of the significance analysis of the environmental factors. The PCOS with phlegm group was mainly concentrated in high BMI, high INS, and androgen. PCOS with nonphlegm-moisture group was mainly concentrated in areas with high androgen, high LH, and low BMI, and the control group was mainly concentrated in areas with low androgen, low BMI, and low INS. This shows that the intestinal flora community structure was quite different between the three groups. Among the top 10 genera with relative abundance, Megamonas was concentrated in areas with high BMI and high INS (1.52, 0.13) (Figure 10).

### 3.5. Functional Changes in Intestinal Flora

**Figure 11:**
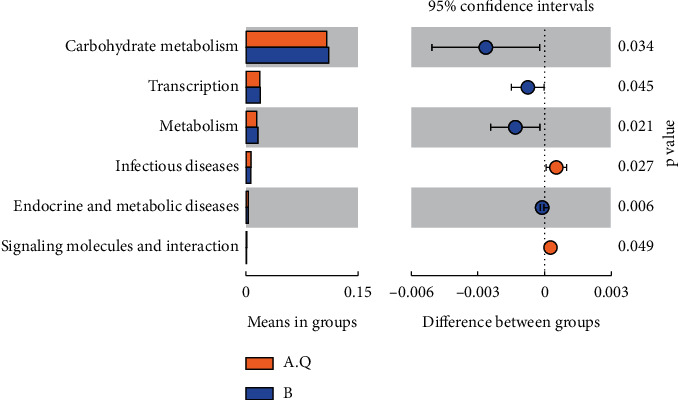
T_test analysis of functional differences between PCOS with phlegm-dampness group and PCOS with nonphlegm-dampness group in the second level.

To further explore the changes in intestinal flora function in patients with PCOS with phlegm-dampness syndrome and PCOS with nonphlegm-dampness syndrome, a predictive analysis of intestinal flora Tax4Fun function was performed. Tax4Fun function prediction was achieved using the nearest neighbor method and based on the smallest 16S rRNA sequence similarity. On the second level, as shown in Figure, the differential functions of A.Q and B groups were carbohydrate metabolism, translation, metabolism, endocrine, and metabolic diseases, signaling molecules and interaction (*P* value <0.05) (Figure 11).

### 3.6. Comparison of Baseline Data between Pretreatment and Posttreatment in PCOS Patients with Phlegm-Dampness Group

There was no significant difference between FSH and PRL before and after treatment in PCOS with phlegm-dampness group (*P* > 0.05); there were differences between BMI, WHR, FPG, FINS, HOMA-IR, LH, and LH/FSH statistical significance (*P* < 0.05). PCOS with phlegm-damp group was lower than before treatment in terms of BMI, waist circumference, hip circumference, WHR, FPG, FINS, and HOMA-IR (*P* < 0.05); T value after treatment was lower than before treatment, but the difference was not statistically significant (*P* > 0.05) (Table 2).

### 3.7. Comparison between Pre- and Post-TCM Treatment in PCOS Patients with Phlegm-Dampness

TCM syndrome scores of PCOS with phlegm-dampness group were found to have improved after treatment, and the difference was statistically significant (*P* < 0.01) (Table 3).

### 3.8. Comparative Analysis of Pre- and Posttreatment Diversity in Intestinal Flora in PCOS Patients with Phlegm-Damp

**Figure 12:**
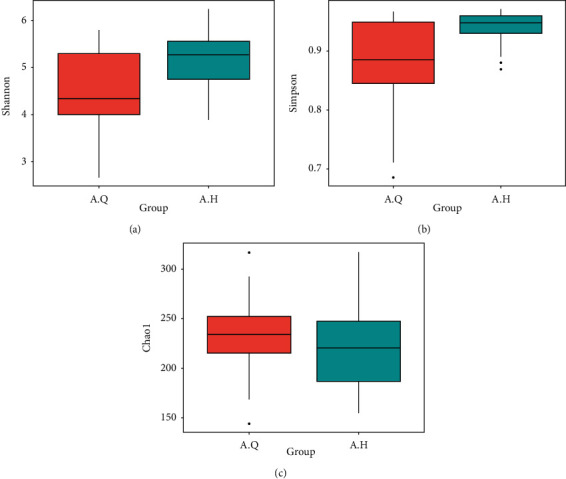
Horizontal axis: A.Q: PCOS with phlegm-dampness syndrome group: intestinal flora before treatment; A.H: intestinal flora of PCOS with phlegm-dampness syndrome group after treatment. Vertical axis: alpha diversity index.

**Figure 13:**
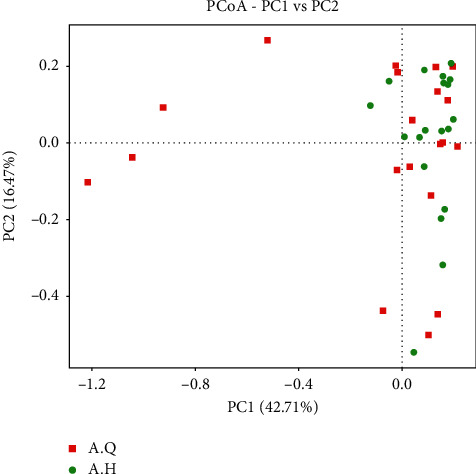
*β* diversity PCoA analysis of weighted UniFrac before and after treatment.

The box diagram illustrating the difference between the groups showed that the Shannon index was greater after the treatment, and the *P* value between the two groups was 0.013, which was considered statistically significant (*P* < 0.05). The Simpson index was greater after treatment, the *P* value between the groups was 0.009, and the difference was statistically significant (*P* < 0.01). The Chao1 index after treatment was less than that before the treatment, the *P* value was 0.242 between the two groups, and the difference was not statistically significant (*P* > 0.05) (Figure 12). Therefore, the diversity of intestinal flora in patients with PCOS with phlegm-dampness syndrome increased after treatment. As shown in the PCoA analysis in Figure 13, the scattered point separation between the pretreatment and posttreatment stool samples in the PCOS with phlegm-dampness syndrome group was sufficient, and some of them were distinguished, indicating that there is a significant difference in *β* diversity after treatment.

### 3.9. Difference in the Composition of Intestinal Flora before and after Treatment

**Figure 14:**
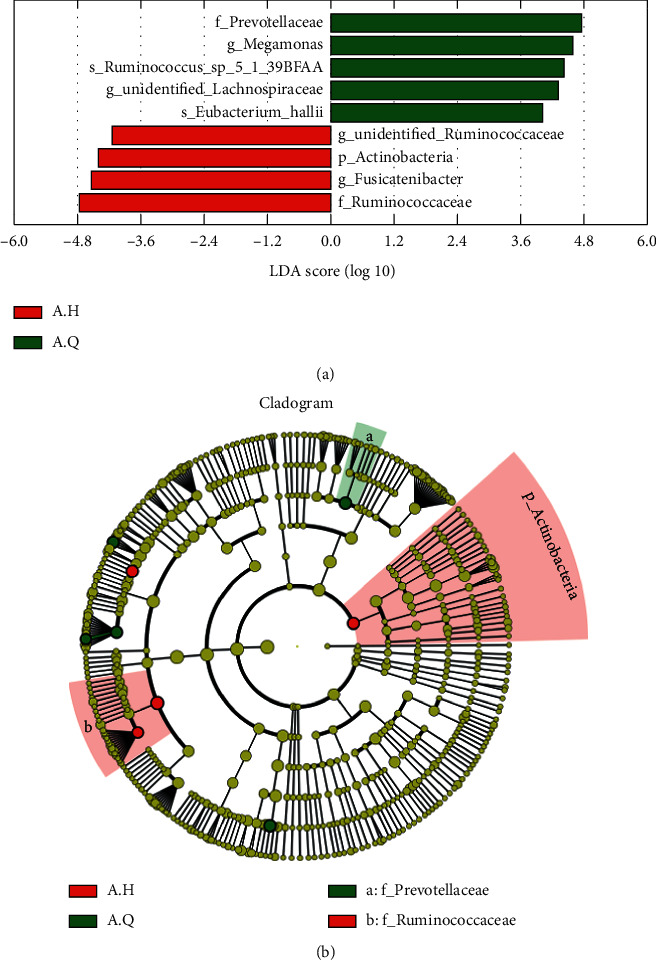
(a) Histogram of species relative abundance at the phylum level before and after treatment (top10); (b) histogram of species relative abundance at the genus level before and after treatment (top10).

**Figure 15:**
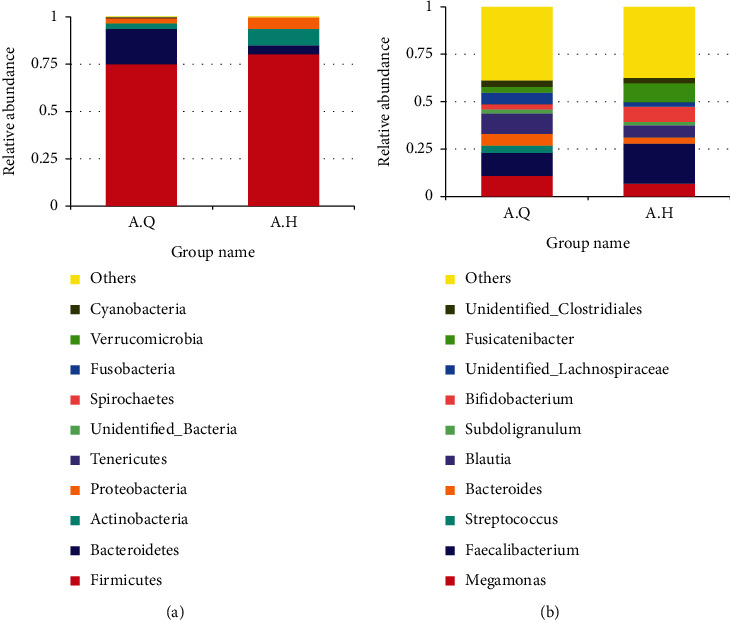
Histogram and evolutionary branching diagram of LDA value distribution of sample flora before and after treatment.

At the phylum level (Figure 14), the colony composition was similar in both pre- and posttreatment. Besides, the top 10 most abundant species were Firmicutes, Bacteroidetes, Actinobacteria, Proteobacteria, Tenericutes, unidentified_Bacteria, Spirochaetes, Fusobacteria, Verrucomicrobia, and Cyanobacteria. Compared with before treatment, the proportions of Firmicutes, Actinobacteria, and Proteobacteria were significantly increased after treatment, while Bacteroidetes was decreased, as shown in Figure 15(a). According to the rank-sum test and LEfSe analysis, at the phylum level, the most enriched species before and after treatment was Actinobacteria. At the genus level, the histogram of the colony composition between groups showed that the top 10 most abundant species were Megamonas, Faecalibacterium, Streptococcus, Bacteroides, Blautia, Subdoligranulum, Bifidobacterium, Unidentified_Lachnospiraceae, Fusicatenibacter, and Unidentified_Clostridiales. As shown in Figure 15(b), the rank-sum test and LEfSe analysis showed that Megamonas and Unidentified_Lachnospiraceae were significantly reduced, while Fusicatenibacter and Unidentified_Ruminococcaceae were significantly increased.

The % content of Bifidobacterium in the top 30 species with the highest abundance at the genus level is as follows: A.Q group: 2.8%; A.H group: 8.2%. At the genus level, the content of Lactobacillus before and after treatment among the top 30 species with the greatest abundance was A.Q group: 0.26%a and A.H group: 1.1%.

### 3.10. Changes in Intestinal Flora before and after Treatment in PCOS Patients with Phlegm-Dampness Syndrome

**Figure 16:**
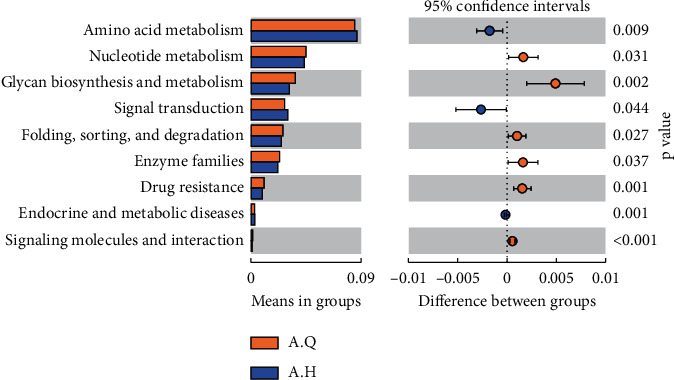
T-test analysis of differential function at level 2 before and after treatment.

Through the prediction of the Tax4Fun function, the changes in intestinal flora in PCOS patients with phlegm-dampness syndrome were analyzed before and after treatment. According to the database annotation results, at the second level (Figure 16), the differential functions that were reported to be higher after treatment than before treatment were involved in amino acid metabolism and signal transduction. Besides, the differential functions that were found to be reduced after treatment were involved in nucleotide metabolism, glycan biosynthesis and metabolism and folding, sorting and degradation, enzyme families, drug resistance, endocrine and metabolic diseases, and signaling molecules and interactions.

## 4. Discussion

PCOS is a multifactorial disorder affecting women's metabolic and reproductive functions. Besides, it seriously affects women's physical and mental health and its etiology is complex. In recent years, the role of intestinal flora in the development of PCOS has become a research hotspot. Chinese medicine believes that phlegm-dampness is the main pathological basis of PCOS, and the main treatment principle is to dry the dampness and reduce phlegm.

In this study, the intestinal flora of the PCOS with phlegm-dampness syndrome group, the PCOS nonphlegm-dampness syndrome group, and the control group was found to be significantly different. The PCOS with phlegm-dampness syndrome group showed a significant imbalance in the intestinal flora. The main associated manifestations were reduced alpha diversity, increased beta diversity, and changes in the structure and composition of the intestinal flora. Studies have found that, compared with healthy women, women with PCOS have lower alpha diversity [14]. Previous studies have also shown that obesity in PCOS patients can cause changes in the composition of the intestinal flora, especially a decrease in alpha diversity [15]. This study found that the number of species (richness) in the PCOS with phlegm-dampness syndrome group was less than in the PCOS with nonphlegm-dampness syndrome group and the control group (*P* < 0.01). The diversity (quantity and uniformity) was less than that in the PCOS nonphlegm-dampness syndrome group and the control group (*P* < 0.05). The alpha diversity index of the PCOS with nonphlegm-dampness syndrome group was slightly higher than that of the control group, but the difference between the two was not statistically significant (*P* > 0.05). This finding was consistent with our experimental results, which showed that the abundance and diversity of intestinal flora in PCOS with phlegm-dampness syndrome group were significantly reduced. Although the relationship between disease and alpha diversity is unclear, there is sufficient amount of literature reporting on alpha diversity in obesity, type 2 diabetes, cardiovascular disease, acute exacerbation of the chronic obstructive pulmonary disease, Crohn's disease, irritable bowel syndromes, and other diseases, which have been reported to decrease significantly.

The content of Bacteroidetes in PCOS patients with phlegm-dampness syndrome was greater than that in PCOS with nonphlegm-dampness syndrome group and the control group. There was no significant difference in Bacteroidetes content between PCOS with nonphlegm-dampness syndrome and the control group. The PCOS patients with phlegm-dampness syndrome had lower Firmicutes compared with PCOS with nonphlegm-dampness group and control group. Besides, the control group contained lower Firmicutes than PCOS with nonphlegm-dampness, but there was no significant difference between the PCOS with nonphlegm-dampness syndrome group and the control group. The content of Actinobacteria was lower in the PCOS with phlegm-dampness group than in the control group. Zeng et al. [16] studied the intestinal flora of patients with PCOS with insulin resistance, patients with PCOS without insulin resistance, and healthy controls and found that the relative abundance of Bacteroides was significantly increased in PCOS patients, especially in PCOS patients with insulin resistance. According to the rank-sum test and LEfSe analysis, there was a significant difference between the three groups in terms of the number of Actinobacteria, and they were found to be more enriched in the control group than in the PCOS with phlegm-dampness syndrome group and the PCOS with nonphlegm-dampness syndrome group. Bacteroides can be subdivided into three broad categories: Bacteroidia, Flavobacteriia, and Sphingobacteriia. Most Bacteroides live in the intestine of humans or animals, but they sometimes become pathogenic bacteria. Previous studies have found [17] a significant increase in some Gram-negative bacteria in the gut of obese PCOS patients. At the genus level, Megamonas was more abundant in the PCOS with phlegm-dampness syndrome group, and the difference was significant. Agathobacter, Bifidobacterium, unidentified Ruminococcaceae, and Anaerostipes were more enriched in the PCOS with phlegm-dampness syndrome group and PCOS with nonphlegm-dampness syndrome group than in the control group. Pseudomonas belongs to the genus Pachyphytum and Clostridia, and it is associated with inflammatory factors throughout the body. It is a proinflammatory bacterium that modifies the occurrence of inflammation in metabolic and mental diseases. Macromonas is significantly increased [18] and a biomarker for type 2 diabetes.

Bifidobacterium belongs to the order Actinobacteria; Lactobacillus belongs to the phylum Firmicutes. The content of Bifidobacterium in the PCOS with phlegm-dampness syndrome group was lower than that in the PCOS with nonphlegm-dampness syndrome group and the control group. The content of Lactobacillus was lower in the PCOS with phlegm-dampness syndrome group than in the PCOS with nonphlegm-dampness syndrome group and the control group. However, Lactobacillus in the PCOS with nonphlegm-dampness syndrome group was more than that in the control group. Previous studies have shown that probiotics can regulate the intestinal ecosystem by promoting the formation of mucus layers, secreting antibacterial factors, the secretion of secreted immunoglobulin A (SLGA), competitive adhesion with intestinal epithelial cells, and the formation of tight junctions [19]. Beneficial bacteria such as Bifidobacteria and lactic acid bacteria can resist infection from pathogenic bacteria, inhibit the growth of harmful bacteria in the human body, synthesize vitamins needed by the human body, promote the absorption of minerals by the human body, produce organic acids to stimulate the peristalsis of the intestines, promote defecation, prevent constipation, and play an important role in purifying the intestinal environment, decomposing carcinogens, stimulating the human immune system, and improving disease resistance. Bifidobacterium is a common probiotic, and some studies have pointed out that, after supplementation with probiotics, metabolic diseases such as type 2 diabetes, obesity, and insulin resistance have been significantly improved, and blood lipid levels reduced [20]. Other studies have found a decrease in the number of Bifidobacteria in the gut of patients with type 2 diabetes, and they believe that impaired glucose tolerance and insufficient insulin secretion in patients with type 2 diabetes are related to a decrease in the number of Bifidobacterium [21]. Lactobacillus is a widely used probiotic, which can ferment carbohydrates to produce lactic acid, prevent harmful bacteria from sticking, help in digestion, and enhance the body's immunity [22]. Naito et al. [23] found that Lactobacillus can improve insulin resistance and improve the state of metabolic disorders in the body. Lactobacillus has also been reported to promote low-level inflammation and increase the occurrence of metabolic endotoxin levels. Lactobacillus has an activating effect on macrophages, making them more cytotoxic. Besides, Lactobacillus can induce human peripheral blood mononuclear cells (PBMCs) to produce IL-12, IL-18, and *γ*-interferon, thereby inducing a Th1-type cellular immune response. It can be inferred that changes in the number of Bifidobacterium and Lactobacillus play a certain role in the occurrence and development of PCOS. However, the specific mechanism needs to be studied further.

In this study, Spearman correlation analysis heat map display and CCA analysis were performed on the clinical parameters of the three groups. Spearman correlation showed that Actinobacteria was negatively correlated with LH, HOMA-IR, and LH/FSH (*P* < 0.05); Proteobacteria was positively correlated with FINS, HOMA-IR, and FPG (*P* < 0.05); Tenericutes was negatively correlated with BMI, FINS, HOMA-IR, and T (*P* < 0.05); and Spirochaetes were positively correlated with FINS, HOMA-IR, and FPG (*P* < 0.05). The higher the insulin resistance was, the greater the number of Proteobacteria and Spirochaetes was, and the smaller the number of Actinobacteria and Tenericutes was. According to CCA analysis, it was found that the PCOS with phlegm-moisture group mainly concentrated in areas with high BMI, high INS, and androgen, while the PCOS with nonphlegm-moisture group mainly concentrated in areas with high androgen, high LH, and low BMI. This shows that the intestinal flora community structure varies between the three groups. Among the top 10 genera with relative abundance, Megamonas was concentrated in areas with high BMI and high INS and in the PCOS with phlegm-dampness group. These findings indicated that the three groups had different microflora structures. These differences, which are influenced by the clinical characteristics of PCOS with phlegm-dampness syndrome, show a clear correlation.

The clinical efficacy of Jiawei Qi Gong Wan prescription in the treatment of PCOS with phlegm-dampness syndrome showed that the levels of BMI, waist circumference, hip circumference, WHR, FPG, FINS, HOMA-IR, and TCM syndrome scores were improved (*P* < 0.05). Besides, after treatment, the alpha diversity of the intestinal flora in this group was higher than that before treatment, and the beta diversity was also significantly different. At the phylum level, the proportion of Firmicutes, Actinobacteria, and Proteobacteria was increased, while Bacteroidetes was decreased after treatment. The species showing a significant difference was Actinobacteria. At the family level, Ruminococcaceae increased significantly and Prevotellaceae decreased significantly after treatment. At the genus level, Megamonas and unidentified_Lachnospiraceae decreased significantly, while Fusicatenibacter and unidentified_Ruminococcaceae increased significantly after treatment. The number of probiotics such as Bifidobacterium and Lactobacillus in the intestinal tract of PCOS patients with phlegm-dampness syndrome was found to be increased after treatment compared with before treatment. Jiawei Qi Gong Wan prescription improved the function of intestinal flora in PCOS patients with phlegm-dampness syndrome. Besides, after treatment, nucleotide metabolism, polysaccharide biosynthesis and metabolism, folding, sorting and degradation, enzyme family, drug resistance, endocrine, and metabolic properties diseases, signal molecules, and interaction functions were found to be reduced. The amino acid metabolism and signal transduction functions were increased after treatment.

The time and funds for this study were limited; hence, the number of clinical cases is small, the results may be one-sided, the observation time short, and a lack of long-term follow-up of drug cases. Therefore, there is a need for a study with larger sample size, to verify the findings. Due to the limited funds available for the experiment, this experiment did not set up PCOS phlegm-dampness syndrome simple western medicine treatment group and traditional Chinese medicine combined with western medicine treatment group in the observation of the efficacy of Qigong pill in the treatment of PCOS phlegm-dampness. Further experiments should be conducted in the future to highlight the advantages of traditional Chinese medicine in the treatment of PCOS with phlegm-dampness syndrome or to reflect on the effects of integrated traditional Chinese and western medicine in the treatment of PCOS phlegm.

In summary, there are some differences in the structure and function of intestinal flora between PCOS patients with phlegm-dampness syndrome and PCOS patients with nonphlegm-dampness syndrome and the control group. The intestinal flora imbalance can be a factor in the pathogenesis of PCOS since it has certain effects on glucose and lipid metabolism, obesity, and the menstrual cycle in PCOS with phlegm-dampness syndrome. Besides, this is related to an increase in lipopolysaccharide bacteria, an increase in proinflammatory bacteria, and a decrease in butyrate-producing bacteria. Chinese medicine Jiawei Qigong Wan prescription increases the intestinal flora diversity of PCOS patients with phlegm-dampness syndrome, changes the structure and functional genes of the intestinal flora, thereby improving their insulin resistance, regulates endocrine metabolism, and improves the symptoms. The intestinal flora interacts with other organs and systems through specific neurotransmitters, hormones, metabolites, and other methods and participates in the regulation of human physiological functions; however, its specific mechanism of action needs to be further explored.

## Figures and Tables

**Table 1:** Anthropometric and metabolic parameters of all participants.

Characteristics	PCOS with phlegm-dampness group (*n* = 20)	PCOS with nonphlegm-dampness group (*n* = 20)	Control group (*n* = 20)	*P* value		
				*P*1	*P*2	*P*3
Age (years)	30.15 ± 3.51	28.85 ± 3.41	29.45 ± 3.61		NS	
BMI (kg/m^2^)	32.23 ± 5.06	22.0 ± 2.17	22.49 ± 3.31	<0.001^*∗∗*^	<0.001^*∗∗*^	1.0
Waist circumference (cm)	88.8 ± 6.16	73.5 ± 4.45	71.25 ± 5.09	<0.001^*∗∗*^	<0.001^*∗∗*^	0.183
Hip circumference (cm)	99.9 ± 2.65	96.5 ± 2.46	95.75 ± 1.83	<0.001^*∗∗*^	<0.001^*∗∗*^	0.315
WHR	0.89 ± 0.04	0.76 ± 0.04	0.74 ± 0.04	<0.001^*∗∗*^	<0.001^*∗∗*^	0.162
FPG (mmol/L)	5.8 ± 0.5	5.39 ± 0.57	5.33 ± 0.45	0.014^*∗*^	0.005^*∗∗*^	0.711
FINS (uU/mL)	22.66 ± 10.66	11.68 ± 5.36	8.78 ± 4.72	0.006^*∗∗*^	<0.001^*∗∗*^	0.346
HOMA-IR	5.94 ± 3.02	2.83 ± 1.45	2.08 ± 1.16	0.004^*∗∗*^	<0.001^*∗∗*^	0.355
FSH (mIU/mL)	6.59 ± 1.27	6.93 ± 0.69	7.06 ± 0.77		NS	
LH (mIU/mL)	10.77 ± 3.15	12.97 ± 2.27	5.61 ± 1.04	0.409	<0.001^*∗∗*^	<0.001^*∗∗*^
LH: FSH ratio	1.66 ± 0.49	1.89 ± 0.35	0.8 ± 0.18	0.558	<0.001^*∗∗*^	<0.001^*∗∗*^
Estradiol (pg/mL)	43.2 ± 9.4	38.4 ± 8.31	38.7 ± 8.46		NS	
Progesterone (ng/mL)	0.46 ± 0.22	0.36 ± 0.21	0.46 ± 0.18		NS	
Prolactin (ng/mL)	11.45 ± 6.62	10.56 ± 5.65	11.88 ± 5.2		NS	
Testosterone (ng/mL)	0.74 ± 0.24	0.61 ± 0.26	0.4 ± 0.14	0.219	<0.001^*∗∗*^	0.017^*∗*^

Data are presented as mean ± SD. Abbreviations: PCOS, polycystic ovary syndrome; BMI, body mass index; WHR, waist-to-hip ratio; FPG, fasting plasma glucose; FINS, fasting plasma insulin; HOMA-IR, homeostasis model assessment of insulin resistance; FSH, follicular stimulating hormone; LH, luteinizing hormone; *P*1, PCOS with phlegm-dampness group vs. PCOS with nonphlegm-dampness group; *P*2, PCOS with phlegm-dampness group vs. control group; *P*3, PCOS with nonphlegm-dampness group vs. control group. NS: there was no significant difference among the three groups, *P* > 0.05; the difference was statistically significant, *P* < 0.05. ^*∗*^There is a significant difference between the two groups (*P*＜0.05); ^*∗∗*^ difference is highly significant (*P*＜0.01).

**Table 2:** Comparison of clinical baseline data before and after treatment in PCOS patients with phlegm-dampness.

Characteristic	PCOS with phlegm-dampness group before treatment (*n* = 20)	PCOS with phlegm-dampness group after treatment (*n* = 20)	*Z* value	*P* value
BMI (kg/m^2^)	32.23 ± 5.06	29.56 ± 3.93	−3.921	*P* < 0.001^*∗∗*^
Waist circumference (cm)	88.8 ± 6.16	82.05 ± 5.09	−3.924	*P* < 0.001^*∗∗*^
Hip circumference (cm)	99.9 ± 2.65	98.3 ± 2.13	−3.335	0.001^*∗∗*^
WHR	0.89 ± 0.04	0.83 ± 0.04	−3.893	*P* < 0.001^*∗∗*^
FPG (mmol/L)	5.8 ± 0.5	5.37 ± 0.35	−3.105	0.002^*∗∗*^
FINS (*μ*U/mL)	22.66 ± 10.66	13.27 ± 5.89	−3.743	*P* < 0.001^*∗∗*^
HOMA-IR	5.94 ± 3.02	3.18 ± 1.47	−3.783	*P* < 0.001^*∗∗*^
FSH (mIU/mL)	6.59 ± 1.27	6.78 ± 0.97	−0.504	0.614
LH (mIU/mL)	10.77 ± 3.15	6.83 ± 2.06	−3.883	*P* < 0.001^*∗∗*^
LH: FSH ratio	1.66 ± 0.49	1.02 ± 0.31	−3.921	*P* < 0.001^*∗∗*^
Prolactin (ng/mL)	11.45 ± 6.62	10.62 ± 4.95	−0.803	0.422
Testosterone (ng/mL)	0.74 ± 0.24	0.65 ± 0.15	−1.195	0.232

Data are presented as mean ± SD. Abbreviation: PCOS, polycystic ovary syndrome; BMI, body mass index; WHR: waist-to-hip ratio; FPG, fasting plasma glucose; FINS, fasting plasma insulin; HOMA-IR, homeostasis model assessment of insulin resistance; FSH, follicular stimulating hormone; LH, luteinizing hormone. ^*∗*^There is a significant difference between the two groups (*P*＜0.05); ^*∗∗*^difference is highly significant (*P*＜0.01).

**Table 3:** Comparison of TCM syndrome points in PCOS with phlegm-dampness group before and after treatment.

Parameters	Before treatment	After treatment	*P* value
TCM syndrome integral	22.05 ± 2.54	10.85 ± 2.06	*P* < 0.001^*∗∗*^

## Data Availability

The data used to support the findings of this study are available from the corresponding author upon request.
